# The Presence of Asthma, the Use of Inhaled Steroids, and Parental Education Level Affect School Performance in Children

**DOI:** 10.1155/2013/762805

**Published:** 2013-07-09

**Authors:** A. Tsakiris, M. Iordanidou, E. Paraskakis, A. Tsalkidis, A. Rigas, S. Zimeras, C. Katsardis, A. Chatzimichael

**Affiliations:** ^1^Michalinio Pediatric Development Centre, Ministry of Employment, Athens, Greece; ^2^Respiratory Unit, Department of Pediatrics, Academic General Hospital of Alexandroupolis, Dragana, Alexandroupolis, Thrace, Greece; ^3^Department of Electrical and Computer Engineering, Democritus University of Thrace, Xanthi, Greece; ^4^Department of Statistics and Actuarial-Financial Mathematics, University of the Aegean, Samos, Greece; ^5^Respiratory Department, General Hospital of Athens “ELPIS,” Athens, Greece

## Abstract

*Objective*. Childhood asthma is a frequent cause of absenteeism that affects school performance. We aimed to investigate the impact of asthma on absenteeism and school performance level of elementary and high school students. *Methods*. Data about sociodemographics, absenteeism, and academic achievement were obtained from 1539 students attending 98 schools in Greece. School performance was assessed for the last two years of school attendance using parents' and teachers' reports and grade point average promotion. *Results*. The mean of the days of absence of students with asthma was higher compared to the healthy students (6.2 ± 11.7 versus 0.3 ± 3.1, resp., *P* < 0.001). Students with reduced healthcare use presented less absenteeism than those with increased healthcare use for asthma (4.3 ± 8.6 versus 12.4 ± 17.0 days, resp., *P* < 0.001). Asthma and healthcare use for asthma accounted for an overall estimated variability in absence days of 13.8% and 9%, respectively. Absenteeism was associated with poor school performance for the last two years of school (*P* = 0.002) and with lower grade point promotion in elementary school students (*P* = 0.001) but not in high school students (*P* = 0.316). Higher level of parental education was associated with better school performance (*P* < 0.001). Asthma was associated with a decreased possibility for excellent performance (OR = 0.64, *P* = 0.049, 95%CI = 0.41–1.00) in elementary students. Students with asthma using inhalers were four times more likely to perform excellently in elementary school (OR = 4.3, *P* = 0.028, 95%CI = 1.17–15.95) than their asthmatic peers with alternative asthma treatments. *Conclusions*. Asthma and increased healthcare use enhance school absenteeism. Inhaled steroid use and the higher parental education level were the most important predicting factors for good school performance in elementary school asthmatic children.

## 1. Introduction

Asthma is the most common chronic childhood disease with increasing prevalence from 31.4 per 1000 population in 1980 to 54.6 per 1000 population in 2000 [1], despite the advances in asthma pathophysiology understanding and treatment. 

Among childhood chronic diseases, asthma is the most frequent cause of school absenteeism. Doull et al. reported that 24% of school students and 55% of asthmatic students missed school days due to respiratory symptoms [2]. School attendance is a normal activity, necessary for a child's social and educational development. School attendance and the limitation of daily activities are both used as indicators of asthma control level in children according to GINA guidelines, C-ACT and ATAQ questionnaires. Indeed, increased absenteeism interrupts learning processes and participation in daily activities. Previous studies have shown that school absenteeism is related to chronic health conditions such as ADHD, diabetes, seizure, and cardiovascular disorders [3]. Moreover it is well known that disabling asthma has profound effects on childrens educational level and functioning [4].

It is widely believed that school absenteeism and poor school performance are increased in students diagnosed as having asthma compared to nonasthmatic students. Although increased absenteeism because of asthma could be a predictor of poor school performance in students with asthma, the impact of asthma on school performance has not been studied sufficiently. Until now, many studies have reported that absenteeism is more frequent in students with asthma compared to nonasthmatic controls [5–7]. However, Millard et al. examined the quantitative effect of asthma on school absenteeism in a sample including children with varying severity of asthma and reported that students with asthma miss no more school days than their classmates without asthma [8].

On the other hand, the results of previous studies regarding the association of absenteeism with school performance are contradictory. Although increased asthma-related absenteeism was associated with poor school performance [5, 9, 10], other studies reported no association between the presence of asthma and school performance, indicating that students with asthma perform as well as their school peers without asthma [5, 7, 11]. This controversy among results could be attributed to the differences in the definitions and diagnosis of asthma, the asthma severity level, and the measures of school performance. 

Our aim was to carry out a cross-sectional study with a sample of students of elementary and high schools in order to examine (1) the impact of the presence of asthma on absenteeism and school performance level as reported by parents, teachers and as recorded according to the students' grade point promotion, (2) the impact of absenteeism on school performance level, (3) the impact of asthma as identified by healthcare use for asthma on absenteeism and school performance level, and (4) other risk factors, except of the presence of asthma, for poor school performance. 

## 2. Methods

### 2.1. Study Design

This study employed a cross-sectional survey of parents of children with asthma. The major goal of this study was to examine the impact of childhood asthma on children's absenteeism and school performance. The study protocol was approved by the Ethics Committee of University Hospital of Alexandroupolis.

Data from 98 schools (75 elementary and 23 high schools) located in the regions Evros, Achaia, and Attiki in Greece was obtained during two consecutive academic years. Initially, a questionnaire about socio-demographics, absenteeism, and academic achievement was provided to the parents/guardians of students through each participating school. The parents/guardians of the students after a written informed consent completed the structured questionnaire. The asthma prevalence is referred to the last two years by the time of the questionnaire. The questionnaire included questions about students' school performance which were answered by teachers. These questions were answered only for students whose parents gave informed written consent for these questions.

Student socio-demographics included gender, age, gestational age and birth weight, parental smoking, in utero exposure to smoking, parental occupation, parental education, and family size. In the asthmatic group, information about received asthma medications, school days of absence during the last two years, asthma-related limitation in daily activities, and frequency of hospital or pediatrician visits because of their asthma was recorded, too. Asthma severity was defined by the number of asthma-related visits to their pediatrician during the last year (reduced healthcare use <5 visits/year and increased healthcare use >5 visits/year).

The average grade score of the students was a continuous value and grade promotion was categorized as excellent, good, average, and bad for all elementary and high schools according to parental reports. Both continuous and categorical achievement outcomes were used as the measure of school performance. School performance was assessed for the last two years of school attendance for each student and grade point average promotion was available for the last two years. Additionally, school performance was based on teachers' reports. Both school absenteeism and school performance data were obtained by parents' reports for the last two years of school attendance, teachers' reports about school performance, and the grade average point promotion for the last two years of school attendance.

### 2.2. Study Population

 Of the 1539 students (47.2% males) who returned the questionnaires, aged 8–16 years old, 262 (17.0%) had been diagnosed as having asthma as reported by their parents/guardians through the question about asthma medications they received in the last two years. Achievement level information was available for 1377 of the students for the last two years.

### 2.3. Statistical Analysis

Analyses were conducted using SPSS 15.0. Personal identifiers were removed prior to analyses to retain anonymity of students participating in this study. Initially, univariate analysis was conducted to determine which variables were significant predictors for academic performance and asthma-related absenteeism. Relative frequencies were calculated for each group and a *χ* ^2^ test analysis was conducted comparing the distribution of these parameters between groups. Data are expressed as mean values with standard deviations (SD) or proportions. Continuous data were tested for normality using the Kolmogorov-Smirnov test of normality. Comparisons for continuous data that do not follow normal distribution in two or three groups were conducted using a Mann-Whitney test or Kruskal-Wallis test, respectively. Factor analysis and principal component analysis (PCA) were used for the calculation of communalities for each variable considering presence or absence of asthma. To estimate the risk of absenteeism associated with the presence of asthma or asthma control level, linear regression analysis was used while to estimate the likelihood of excellent school performance with the presence of asthma, the use of inhaled corticosteroids, and the parental education, binary logistic regression was performed. All *P* values are two sided with a 0.05 type I error and 95% power. A value of *P* < 0.05 was considered statistically significant.

## 3. Results

A total of 1539 students, aged 8–16 years old, were enrolled in this study. 262 (17.0%) of the students had asthma and 727 (47.2%) were males. The grade level distribution was 997 students in elementary school and 540 students in high school. All sociodemographic characteristics of students with or without asthma are shown in Tables 1 and 2. Calculations of the communalities for each variable considering presence or absence of asthma are depicted in Table 3. Based on the multivariate analysis we proposed 5 clusters (factors *F*_*i*_) for asthma group, which explain the 80.94% of the initial information and 4 clusters for no asthma group, which explain the 78.39% of the initial information. These factors for the asthmatic group are factor 1: maternal smoking, in utero smoking, factor 2: absence days, days of continuous absence, factor 3: brothers/sisters, birth order, factor 4: father education, mother education, and factor 5: first school performance, final school performance, and final grade elementary.

**Figure 1:**
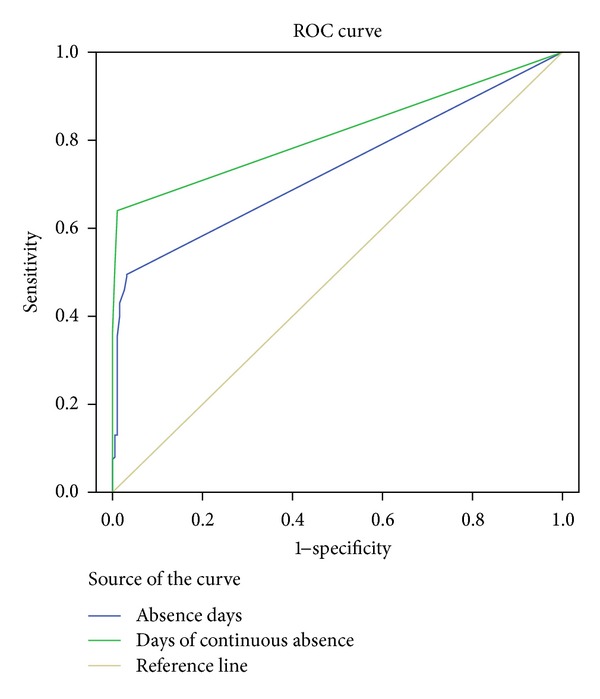
ROC curve for asthmatic students based on their absences in school (days of absence and days of continuous absence).

The factors for the nonasthmatic group are factor 1: maternal smoking, in utero smoking, factor 2: absence days, days of continues absence, factor 3: brothers/sisters, birth order, and factor 4: first school performance, final school performance, and final grade elementary.  Figure 1 illustrates the graphical presentation of the sensitivity using ROC curves for the absence days and days of continuous absence considering the presence of asthma diagnosis with accuracy 0.734 and 0.817, respectively.

### 3.1. Absenteeism

Regarding absenteeism during the last two academic years, the mean days of absence were 1.6 ± 6.5 days when all students combined. The highest mean absenteeism was among elementary school students (1.8 ± 7.0 days), significantly different when compared to high school students' absent days (1.2 ± 5.6) (*P* < 0.001). Additionally, when students were divided according to the presence of asthma, students with asthma were absent significantly more days than nonasthmatic control students. Particularly, the mean days absence of students with asthma were 6.2 ± 11.7 days, significantly higher than 0.3 ± 3.1 recorded for nonasthmatic healthy control students (*P* < 0.001) (Table 4). Information about asthma control level was available for 243 out of 262 asthmatic students. Among asthmatic children, absenteeism increased according to asthma severity as defined by number of asthma-related visits to hospital or pediatrician yearly; increased health care use (12.4 ± 17.0 days) versus reduced health care use (4.3 ± 8.6 days) (*P* < 0.001). Using linear regression analysis, children with asthma were significantly more likely than those without asthma to have been absent from school more days (*P* < 0.001). Similarly, in the linear regression analysis comparing healthcare use for asthma and school days missed among asthmatic students, mean days absence increased with increased healthcare use (*P* < 0.001). Using *R* ^2^ change test, we found that the presence of asthma accounts for an estimated 13.8% of overall variability in absence days and asthma control level accounts for an estimated 9% of overall variability in absence days.

### 3.2. School Performance

For school performance, data were available for 1530 students about school performance for the last two academic years according to parental report and for 837 elementary and 540 high school students for grade point promotion for the last two academic years. Additionally, teachers' report about students' school performance was available for 268 students. For the last two years of school achievement, 674 students had excellent performance, 572 students had very good performance, 268 students had good performance, and 16 students had poor performance. 

Students without asthma performed significantly better than students with asthma for the last two years of school attendance in elementary school but not in high school. Particularly, in elementary school students, 59.0% of students without asthma performed excellently during the last two years compared to 43.3% of students with asthma (*P* = 0.003) (Table 4). Furthermore, excellent performance was reported for the 42.2% of students without asthma and the 33.3% of students with asthma based on teachers' report (*P* = 0.034) (Table 4). According to grade point promotion, excellent performance was reported for 85.6% of children without asthma and 79.2% of children with asthma (*P* = 0.047) in elementary schools while no association was found for high school students (*P* = 0.786). Among children with asthma, excellent performance for the last two academic years was reported for 81.2% of children who received inhalers and for 50.0% of children who did not receive inhalers for asthma therapy in elementary schools (*P* = 0.018) (Table 5), while a similar association was not reported in high school students (data not shown).

We examined the association of absenteeism with the three measures of school functioning used in this study. In the total of students, absenteeism was associated with poor school performance for the last two years of school (*P* = 0.002). Although absenteeism was associated with lower grade point promotion in elementary school students (*P* = 0.001), this association was not verified in high school students (*P* = 0.316). 

Additionally, parental education was found to be significantly associated with school performance. For all students combined, higher level both of paternal and maternal education was significantly associated with better school performance for the last two years of education (*P* < 0.001 and *P* < 0.001, resp.) and better grade point promotion both for elementary school students (*P* < 0.001 and *P* < 0.001, resp.) and high school students (*P* < 0.001 and *P* < 0.001, resp.). When students were classified according to the presence of asthma, all these associations remained statistically significant both for asthmatic and nonasthmatic healthy control students. Furthermore, the higher level of paternal education was negatively associated with the presence of asthma (*P* = 0.010).

Finally, binary logistic regression analysis showed that for the two last years of school performance, the presence of asthma was associated with a decreased possibility for excellent school performance (OR = 0.64, *P* = 0.049, 95%CI = 0.41–1.00) in elementary school students, while a similar association was not found in high school students. Regarding the use of inhalers in asthmatic students, asthmatic students who used inhalers were four times more likely to perform excellently in elementary school (OR = 4.3, *P* = 0.028, 95%CI = 1.17–15.95) than asthmatic elementary school students who did not receive inhalers for asthma therapy. However, this association was not verified in high school students. 

## 4. Discussion

In our study, we examined the association of absenteeism and presence of asthma with school performance in elementary and middle school students. Our main findings are that school absenteeism is higher in students with asthma compared to students without asthma and is associated with increased healthcare use. Furthermore, school absenteeism and the presence of asthma were significantly correlated with poor school performance in elementary school students. Additionally, the novel finding in our study is that lower parental education is a significant contributing factor of poor school performance, too. Regarding the association of antiasthmatic therapy with school performance among asthmatic children, the use of inhalers was associated with excellent school performance only in elementary school children.

There have been few previous studies of the effect of asthma on school absenteeism. Fowler et al. reported that asthmatic children had a higher prevalence of grade failure, learning disabilities, and absenteeism [10]. A possible mechanism for how asthma could lead to poor school performance is the higher absenteeism that is associated with decreased school attendance. Our measures of school performance were based not only on parental reports but they were also validated with grade point promotion and teachers' reports. Indeed, our results are in accordance with other studies that have been carried out so far suggesting that school absenteeism is a significant contributing factor that determines school performance. According to our analyses for the association of absenteeism with school performance, as the number of absence days increases, grade point average promotion for the last two years decreases. Furthermore, students with more severe asthma had more school missed days compared to students with mild asthma suggesting that not only the presence of asthma but also the level of healthcare use contributes to school attendance and consequently to school performance. However, this association was not verified in high school students. Nevertheless, the association between school absenteeism and school performance is still unclear. The discrepancies reported among other studies about school performance and school absenteeism could be attributed to the lack of information about asthma severity that was not taken into account in previous analyses. 

In this study the identification of children with asthma was based only on parental reports that have been demonstrated to be valid for chronic conditions [12]. However, in this case the prevalence of asthma may be under- or overestimated due to undiagnosed or misdiagnosed cases. The prevalence of asthma in our population of elementary and middle school children is higher than that reported in other studies. The estimated incidence of lifetime childhood asthma in Greece is 7–12.6% according to other published studies [12, 13]. This difference could be attributed to the overestimation of asthma diagnosis by parents or the higher prevalence of asthma in Attiki and Achaia. Furthermore, the prevalence of childhood asthma is higher among young children and in large urban areas; 65% of our population was enrolled in elementary schools and in this study two of the three larger areas in Greece participated. 

Additionally, asthma severity assessment in this study was based on the number of visits to hospital or pediatrician during last year because of asthma. Actually previous studies have shown that children with severe asthma visit more frequently their pediatrician or the hospital mainly due to exacerbation in order to obtain the appropriate management [14]. A limitation in our study is that clinical measurements of asthma severity, including spirometric or FeNO values, are not available and we were unable to evaluate the asthma control level using these parameters. Another limitation that should be mentioned is the lack of detailed information about the use of asthma medications since, in this study, asthma treatment assessment was based on parental reports about the use of asthma medication.

Nevertheless, we found that the use of inhalers was significantly associated with excellent school performance only in elementary school children. Administration of inhaled corticosteroids, the most effective therapy for asthma treatment, may reduce and control asthma symptoms leading to lower school absenteeism and consequently to improved school attendance and performance. 

Our findings pointed out parental education level seems to be an important factor affecting absenteeism and school performance. A number of studies have not taken this factor in account and this may be one of the reasons why the discrepancy of the results coming from the studies of school performance of asthmatic children exists.

## 5. Conclusions

In conclusion, consistent with other findings, our results suggest that presence of asthma is associated with school performance and absenteeism. Students with asthma are absent more often and present poorer school performance than students without asthma. Furthermore, we found an association between the frequency of healthcare use and absenteeism. The novel finding is that the use of inhalers is another independent factor predicting better school performance in elementary schools among asthmatic students. The use of inhalers affects asthma control level and consequently the learning process and the school performance. Finally, the parental education level can predict school absenteeism and performance both in elementary and high school students and it should be taken in account in future studies on that field. 

## Figures and Tables

**Table 1:** Characteristics of students with asthma and nonasthmatic control subjects.

Characteristics	Students with asthma	Nonasthmatic control students	*P* value
Gender *n* (%)			
Male	147 (56.1)	580 (45.4)	0.002
Female	115 (43.9)	697 (54.6)	
Birth order *n* (%)			
1	123 (46.9)	640 (50.1)	0.639
2	97 (37.0)	470 (36.8)	
3	28 (10.7)	117 (9.2)	
4	12 (4.6)	39 (3.1)	
≥5	2 (0.8)	11 (0.9)	
In utero exposure to smoking *n* (%)			
Yes	221 (84.4)	1140 (89.3)	0.021
No	41 (15.6)	136 (10.7)	
Gestational age (weeks) *n* (%)			
<36	65 (25.5)	303 (24.4)	0.494
36–40	176 (69.0)	853 (68.8)	
>40	14 (5.5)	84 (6.8)	

**Table 2:** Family characteristics of students with asthma and nonasthmatic control subjects.

Characteristics	Students with asthma *n* (%)	Nonasthmatic control students *n* (%)	*P* value
Paternal education (years)			
0	2 (0.8)	4 (0.3)	0.010
6	40 (15.3)	144 (11.3)	
6–9	66 (25.2)	252 (19.8)	
9–12	91 (34.7)	479 (37.7)	
University level	42 (16.0)	201 (15.8)	
Master, PhD	21 (8.0)	191 (15.0)	
Paternal smoking			
Yes	135 (51.5)	717 (56.1)	0.144
No	127 (48.5)	560 (43.9)	
Maternal education (years)			
0	2 (0.8)	3 (0.2)	0.132
≤6	33 (12.6)	116 (9.1)	
6–9	51 (19.5)	207 (16.2)	
9–12	103 (39.3)	557 (43.8)	
University level	46 (17.6)	221 (17.4)	
Master, PhD	27 (10.3)	170 (13.4)	
Maternal smoking			
Yes	115 (43.9)	505 (39.5)	0.191
No	147 (56.1)	772 (60.5)	
Family status			
Engaged	263 (85.1)	1150 (90.1)	0.004
Divorced	34 (11.0)	86 (6.7)	
Widowed	8 (2.6)	37 (2.9)	
Single parent	4 (1.3)	3 (0.2)	

**Table 3:** Calculation of the communalities for each variable using PCA.

Variables	Asthmatic	Nonasthmatic
Maternal smoking	0.724	0.719
In utero smoking	0.725	0.718
Absence days	0.766	
Days of continuous absence	0.826	
Brothers/sisters	0.8	0.792
Birth order	0.845	0.791
Father education	0.853	0.788
Mother education	0.817	0.782
First school performance	0.8	0.784
Final school performance	0.89	0.877
Final grade elementary	0.857	0.804

**Table 4:** Absenteeism and school functioning in asthmatic and nonasthmatic students.

	Nonasthmatic students	Asthmatic students	*P* value
Absence days (mean ± SD)	0.3 ± 3.1	6.2 ± 11.7	<0.001
School performance in last two years			
Excellent *n* (%)	575 (45.3)	99 (37.9)	0.127
Very good *n* (%)	467 (36.9)	104 (39.8)	
Good *n* (%)	214 (16.9)	54 (20.7)	
Bad *n* (%)	12 (0.9)	4 (1.5)	
Grade point average for elementary school mean (±SD) in last two years			
<8 *n* (%)	98 (14.4)	33 (20.8)	0.047
>8 *n* (%)	582 (85.6)	126 (79.2)	
Grade point level for middle school in last two years *n* (%)			
<18.5 *n* (%)	343 (73.0)	50 (71.4)	0.786
>18.5 *n* (%)	127 (27.0)	20 (28.6)	
Teachers' report for school performance			
Excellent *n* (%)	10 (6.0)	3 (2.9)	0.034
Very good *n* (%)	41 (24.7)	20 (19.6)	
Good *n* (%)	45 (27.1)	45 (44.1)	
Bad *n* (%)	70 (42.2)	34 (33.4)	

**Table 5:** School performance according to school absenteeism, parental education level, use of inhaled corticosteroids, and number of visits to hospital or pediatrician yearly in elementary school asthmatic students.

	Grade point level	Grade point level	*P* value
	<8	>8	
Absence days (mean ± SD)	2.6 ± .3.8	5.2 ± 8.4	0.183
Paternal education level			
0 *n* (%)	2 (6.1)	0 (0.0)	0.003
6 *n* (%)	11 (33.3)	18 (4.3)	
6–9 *n* (%)	10 (30.3)	30 (23.8)	
9–12 *n* (%)	7 (21.2)	46 (36.5)	
University level *n* (%)	2 (6.1)	22 (17.5)	
Master, PhD *n* (%)	1 (3.0)	10 (7.9)	
Maternal education level			
0 *n* (%)	1 (3.0)	0 (0.0)	0.008
6 *n* (%)	10 (30.3)	14 (11.1)	
6–9 *n* (%)	9 (27.3)	24 (19.0)	
9–12 *n* (%)	9 (27.3)	51 (40.5)	
University level *n* (%)	3 (9.1)	21 (16.7)	
Master, PhD *n* (%)	1 (3.0)	16 (12.7)	
Use of inhaled corticosteroids (yes) *n* (%)	28 (84.8)	121 (96.0)	0.018
Number of pediatrician asthma visits (yearly) *n* (%)			
<5 *n* (%)	19 (63.3)	87 (75.0)	0.202
>5 *n* (%)	11 (36.7)	29 (25.0)	
